# Poly[bis­[chlorido­cop­per(I)]-μ_4_-1,4-bis­[1-(3-pyridylmeth­yl)-1*H*-benzimid­azol-2-yl]butane]

**DOI:** 10.1107/S1600536808008696

**Published:** 2008-04-10

**Authors:** Wei-Ping Zhang, Ying-Ying Liu, Jian-Fang Ma

**Affiliations:** a Department of Chemistry, Northeast Normal University, Changchun 130024, People’s Republic of China; b Jilin Medical College, Jilin 132013, People’s Republic of China

## Abstract

The title Cu^I^ coordination polymer, [Cu_2_Cl_2_(C_30_H_28_N_6_)]_*n*_, was obtained by reaction of CuCl_2_·2H_2_O and 1,4-bis­[1-(3-pyridyl­meth­yl)-1*H*-benzimidazol-2-yl]butane. Each Cu^I^ cation is three-coordinated by a ClN_2_ donor set. The anion acts as a tetra­dentate ligand, linking Cu^I^ centres into a polymeric chain.

## Related literature

For a related compound, see Wang & Xu (2007 ▶). For details of the synthesis, see: Li *et al.* (2007 ▶).
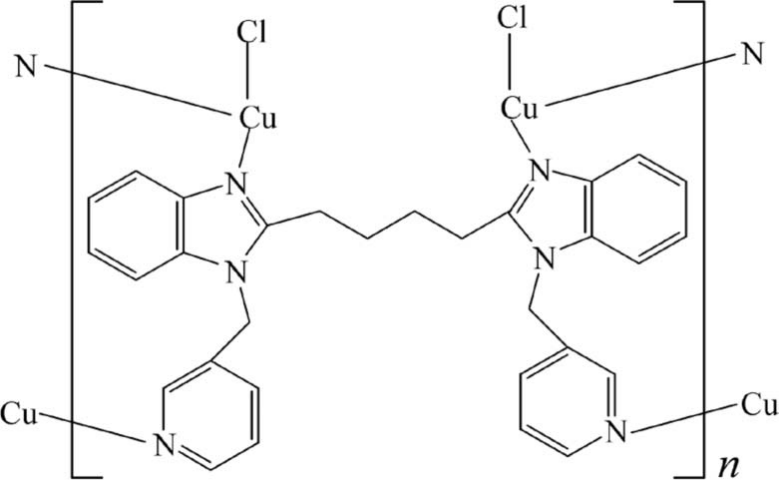


## Experimental

### 

#### Crystal data

[Cu_2_Cl_2_(C_30_H_28_N_6_)]
*M* *_r_* = 670.56
Monoclinic, 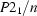

*a* = 9.4792 (11) Å
*b* = 17.810 (2) Å
*c* = 17.326 (2) Å
β = 97.412 (2)°
*V* = 2900.5 (6) Å^3^
*Z* = 4
Mo *K*α radiation
μ = 1.68 mm^−1^
*T* = 293 (2) K
0.23 × 0.20 × 0.09 mm

#### Data collection

Bruker APEX CCD area-detector diffractometer
Absorption correction: multi-scan (*SADABS*; Sheldrick, 1996 ▶) *T*_min_ = 0.66, *T*_max_ = 0.84
17514 measured reflections
6759 independent reflections
3170 reflections with *I* > 2σ(*I*)
*R*_int_ = 0.062

#### Refinement

*R*[*F* ^2^ > 2σ(*F* ^2^)] = 0.054
*wR*(*F* ^2^) = 0.185
*S* = 0.97
6759 reflections
361 parameters
H-atom parameters constrained
Δρ_max_ = 0.42 e Å^−3^
Δρ_min_ = −0.43 e Å^−3^

### 

Data collection: *SMART* (Bruker, 1999 ▶); cell refinement: *SAINT* (Bruker, 1999 ▶); data reduction: *SAINT*; program(s) used to solve structure: *SHELXS97* (Sheldrick, 2008 ▶); program(s) used to refine structure: *SHELXL97* (Sheldrick, 2008 ▶); molecular graphics: *SHELXTL-Plus* (Sheldrick, 2008 ▶); software used to prepare material for publication: *SHELXL97*.

## Supplementary Material

Crystal structure: contains datablocks global, I. DOI: 10.1107/S1600536808008696/bt2684sup1.cif

Structure factors: contains datablocks I. DOI: 10.1107/S1600536808008696/bt2684Isup2.hkl

Additional supplementary materials:  crystallographic information; 3D view; checkCIF report

## Figures and Tables

**Table:** 

Cu1—N4	1.940 (4)
Cu1—N6^i^	2.079 (5)
Cu1—Cl1	2.1836 (16)
Cu2—N1	1.942 (4)
Cu2—N5^ii^	2.116 (4)
Cu2—Cl2	2.1672 (16)

**Table:** 

N4—Cu1—N6^i^	111.23 (18)
N4—Cu1—Cl1	141.19 (13)
N6^i^—Cu1—Cl1	107.12 (13)
N1—Cu2—N5^ii^	108.71 (17)
N1—Cu2—Cl2	142.93 (13)
N5^ii^—Cu2—Cl2	107.36 (13)

Symmetry codes: (i) 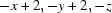; (ii) 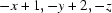.

**Fig. 1.**
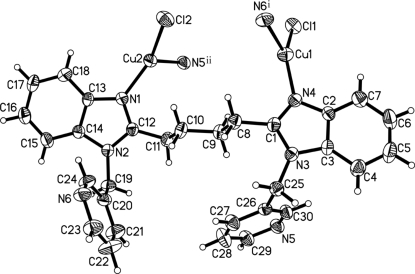
A view of the title compound. Displacement ellipsoids are drawn at the 30% probability level. [Symmetry codes: (i) -x + 2, -y + 2, -z; (ii) -x + 1, -y + 2, -z.]

## supplementary crystallographic information

### Comment

As shown in Fig. 1, three are two crystallographically unique Cu ions, and each Cu^I^ ion is three-coordinated by one Cl^-^ anion and two nitrogen atoms from two *L* anions. Each *L* anion in (I) coordinates to four Cu^I^ cations through its two imidazole N atoms and two pyridine N atoms, thus acing as a tetradentate ligand. The Cu^I^ cations are linked by *L* anions to form a chain along *a* axis. For a related compound, see Wang & Xu (2007).

### Experimental

The ligand was synthesized according to the literature (Li *et al.*, 2007) but 4-(chloromethyl)pyridine was replaced by 3-(chloromethyl)pyridine. A mixture of CuCl_2_.2H_2_O (0.034 g, 2 mmol), H_2_L (0.945 g, 2 mmol), and water (8 ml) was sealed in a Teflon reactor (15 ml) and heated at 130 °C for 3 days. After the mixture had been cooled to room temperature at 10 °C.h^-1^, red crystals of the title compound were obtained.

### Refinement

All H-atoms bound to carbon were refined using a riding model with d(C—H) = 0.93 Å, *U*_iso_=1.2*U*_eq_ (C) for aromatic and 0.97 Å, *U*_iso_ = 1.5*U*_eq_ (C) for CH_2_ atoms.

### Crystal data

**Table:**

[Cu_2_Cl_2_(C_30_H_28_N_6_)]	*F*_000_ = 1368
*M**_r_* = 670.56	*D*_x_ = 1.536 Mg m^−^^3^
Monoclinic, *P*2_1_/*n*	Mo *K*α radiation λ = 0.71073 Å
Hall symbol: -P 2yn	Cell parameters from 3170 reflections
*a* = 9.4792 (11) Å	θ = 1.7–28.3º
*b* = 17.810 (2) Å	µ = 1.68 mm^−^^1^
*c* = 17.326 (2) Å	*T* = 293 (2) K
β = 97.412 (2)º	Block, red
*V* = 2900.5 (6) Å^3^	0.23 × 0.20 × 0.09 mm
*Z* = 4	

### Data collection

**Table:**

Bruker APEX CCD area-detector diffractometer	6759 independent reflections
Radiation source: fine-focus sealed tube	3170 reflections with *I* > 2σ(*I*)
Monochromator: graphite	*R*_int_ = 0.062
*T* = 293(2) K	θ_max_ = 28.3º
ω scans	θ_min_ = 1.7º
Absorption correction: multi-scan(SADABS; Sheldrick, 1996)	*h* = −11→12
*T*_min_ = 0.66, *T*_max_ = 0.84	*k* = −19→23
17514 measured reflections	*l* = −22→22

### Refinement

**Table:**

Refinement on *F*^2^	Secondary atom site location: difference Fourier map
Least-squares matrix: full	Hydrogen site location: inferred from neighbouring sites
*R*[*F*^2^ > 2σ(*F*^2^)] = 0.054	H-atom parameters constrained
*wR*(*F*^2^) = 0.186	*w* = 1/[σ^2^(*F*_o_^2^) + (0.0872*P*)^2^] where *P* = (*F*_o_^2^ + 2*F*_c_^2^)/3
*S* = 0.97	(Δ/σ)_max_ = 0.001
6759 reflections	Δρ_max_ = 0.42 e Å^−^^3^
361 parameters	Δρ_min_ = −0.43 e Å^−^^3^
Primary atom site location: structure-invariant direct methods	Extinction correction: none

### Special details

**Table:**

Geometry. All e.s.d.'s (except the e.s.d. in the dihedral angle between two l.s. planes) are estimated using the full covariance matrix. The cell e.s.d.'s are taken into account individually in the estimation of e.s.d.'s in distances, angles and torsion angles; correlations between e.s.d.'s in cell parameters are only used when they are defined by crystal symmetry. An approximate (isotropic) treatment of cell e.s.d.'s is used for estimating e.s.d.'s involving l.s. planes.
Refinement. Refinement of *F*^2^ against ALL reflections. The weighted *R*-factor *wR* and goodness of fit *S* are based on *F*^2^, conventional *R*-factors *R* are based on *F*, with *F* set to zero for negative *F*^2^. The threshold expression of *F*^2^ > σ(*F*^2^) is used only for calculating *R*-factors(gt) *etc*. and is not relevant to the choice of reflections for refinement. *R*-factors based on *F*^2^ are statistically about twice as large as those based on *F*, and *R*- factors based on ALL data will be even larger.

### Fractional atomic coordinates and isotropic or equivalent isotropic displacement parameters (Å^2^)

**Table:**

	*x*	*y*	*z*	*U*_iso_*/*U*_eq_	
Cu1	0.74531 (8)	0.80687 (4)	0.14462 (4)	0.0609 (2)	
Cu2	0.78422 (7)	0.87957 (4)	−0.16694 (4)	0.0574 (2)	
C1	0.7866 (5)	0.9707 (3)	0.1746 (3)	0.0456 (12)	
C2	0.7040 (5)	0.9140 (3)	0.2683 (3)	0.0460 (12)	
C3	0.7232 (5)	0.9888 (3)	0.2904 (3)	0.0474 (12)	
C4	0.6930 (6)	1.0155 (4)	0.3612 (3)	0.0660 (16)	
H4	0.7093	1.0652	0.3764	0.079*	
C5	0.6365 (7)	0.9631 (4)	0.4082 (3)	0.083 (2)	
H5	0.6131	0.9783	0.4563	0.099*	
C6	0.6141 (7)	0.8894 (4)	0.3856 (3)	0.081 (2)	
H6	0.5730	0.8567	0.4181	0.097*	
C7	0.6507 (6)	0.8627 (3)	0.3163 (3)	0.0646 (16)	
H7	0.6399	0.8123	0.3026	0.078*	
C8	0.8373 (6)	0.9869 (3)	0.0976 (3)	0.0552 (14)	
H8A	0.8916	0.9442	0.0828	0.066*	
H8B	0.9003	1.0300	0.1033	0.066*	
C9	0.7150 (5)	1.0027 (3)	0.0326 (3)	0.0493 (12)	
H9A	0.6547	0.9587	0.0243	0.059*	
H9B	0.6577	1.0438	0.0479	0.059*	
C10	0.7729 (5)	1.0226 (3)	−0.0425 (3)	0.0581 (14)	
H10A	0.8318	0.9817	−0.0568	0.070*	
H10B	0.8325	1.0669	−0.0338	0.070*	
C11	0.6556 (6)	1.0379 (3)	−0.1096 (3)	0.0559 (14)	
H11A	0.5821	1.0000	−0.1093	0.067*	
H11B	0.6130	1.0863	−0.1016	0.067*	
C12	0.7078 (5)	1.0375 (3)	−0.1868 (3)	0.0466 (12)	
C13	0.8090 (5)	1.0002 (3)	−0.2879 (3)	0.0452 (12)	
C14	0.7697 (5)	1.0753 (3)	−0.3012 (3)	0.0499 (13)	
C15	0.7930 (7)	1.1126 (3)	−0.3687 (3)	0.0685 (17)	
H15	0.7635	1.1619	−0.3781	0.082*	
C16	0.8612 (8)	1.0735 (4)	−0.4208 (4)	0.082 (2)	
H16	0.8805	1.0972	−0.4661	0.099*	
C17	0.9024 (7)	0.9993 (4)	−0.4079 (3)	0.0748 (19)	
H17	0.9484	0.9745	−0.4447	0.090*	
C18	0.8765 (6)	0.9618 (3)	−0.3416 (3)	0.0589 (14)	
H18	0.9038	0.9119	−0.3334	0.071*	
C19	0.6695 (6)	1.1750 (3)	−0.2180 (3)	0.0558 (14)	
H19A	0.5894	1.1755	−0.1883	0.067*	
H19B	0.6417	1.2019	−0.2663	0.067*	
C20	0.7944 (6)	1.2145 (3)	−0.1722 (3)	0.0521 (13)	
C21	0.7711 (7)	1.2767 (4)	−0.1289 (4)	0.0792 (19)	
H21	0.6790	1.2932	−0.1258	0.095*	
C22	0.8856 (9)	1.3143 (4)	−0.0902 (4)	0.095 (2)	
H22	0.8707	1.3572	−0.0617	0.114*	
C23	1.0253 (7)	1.2885 (4)	−0.0931 (4)	0.0750 (18)	
H23	1.1017	1.3144	−0.0664	0.090*	
C24	0.9395 (6)	1.1933 (3)	−0.1734 (3)	0.0620 (15)	
H24	0.9582	1.1526	−0.2042	0.074*	
C25	0.7993 (5)	1.1051 (3)	0.2233 (3)	0.0497 (13)	
H25A	0.8834	1.1140	0.1981	0.060*	
H25B	0.8160	1.1266	0.2751	0.060*	
C26	0.6735 (5)	1.1438 (3)	0.1775 (3)	0.0447 (12)	
C27	0.6887 (6)	1.1899 (3)	0.1157 (3)	0.0600 (15)	
H27	0.7789	1.2003	0.1027	0.072*	
C28	0.5706 (6)	1.2209 (4)	0.0727 (4)	0.0742 (18)	
H28	0.5800	1.2532	0.0315	0.089*	
C29	0.4405 (6)	1.2033 (3)	0.0918 (3)	0.0619 (15)	
H29	0.3606	1.2231	0.0619	0.074*	
C30	0.5386 (6)	1.1306 (3)	0.1931 (3)	0.0533 (13)	
H30	0.5270	1.0999	0.2352	0.064*	
N1	0.7676 (4)	0.9779 (2)	−0.2158 (2)	0.0484 (10)	
N2	0.7044 (4)	1.0974 (2)	−0.2354 (2)	0.0491 (11)	
N3	0.7773 (4)	1.0246 (2)	0.2295 (2)	0.0459 (10)	
N4	0.7451 (4)	0.9039 (2)	0.1955 (2)	0.0489 (10)	
N5	0.4217 (5)	1.1587 (3)	0.1516 (3)	0.0584 (12)	
N6	1.0488 (5)	1.2277 (2)	−0.1335 (3)	0.0610 (12)	
Cl1	0.60349 (16)	0.71402 (8)	0.10540 (9)	0.0659 (4)	
Cl2	0.93594 (16)	0.80208 (9)	−0.10598 (10)	0.0758 (5)	

### Atomic displacement parameters (Å^2^)

**Table:**

	*U*^11^	*U*^22^	*U*^33^	*U*^12^	*U*^13^	*U*^23^
Cu1	0.0632 (5)	0.0542 (4)	0.0644 (5)	0.0018 (3)	0.0055 (4)	−0.0063 (3)
Cu2	0.0579 (5)	0.0523 (4)	0.0641 (4)	0.0078 (3)	0.0158 (3)	0.0094 (3)
C1	0.033 (3)	0.057 (3)	0.047 (3)	0.005 (2)	0.007 (2)	0.002 (3)
C2	0.040 (3)	0.050 (3)	0.049 (3)	0.002 (2)	0.007 (2)	0.008 (2)
C3	0.048 (3)	0.057 (3)	0.038 (3)	−0.005 (2)	0.007 (2)	0.002 (2)
C4	0.074 (4)	0.071 (4)	0.053 (3)	−0.007 (3)	0.009 (3)	−0.007 (3)
C5	0.100 (5)	0.105 (6)	0.047 (3)	−0.014 (4)	0.019 (3)	−0.004 (4)
C6	0.098 (6)	0.088 (5)	0.058 (4)	−0.019 (4)	0.014 (4)	0.017 (4)
C7	0.071 (4)	0.059 (4)	0.062 (4)	−0.011 (3)	0.000 (3)	0.001 (3)
C8	0.053 (3)	0.061 (3)	0.053 (3)	−0.002 (3)	0.013 (3)	−0.002 (3)
C9	0.049 (3)	0.059 (3)	0.040 (3)	−0.002 (3)	0.007 (2)	0.000 (2)
C10	0.051 (3)	0.076 (4)	0.051 (3)	−0.004 (3)	0.018 (3)	−0.003 (3)
C11	0.058 (4)	0.065 (4)	0.047 (3)	0.006 (3)	0.016 (3)	0.001 (3)
C12	0.043 (3)	0.051 (3)	0.046 (3)	0.000 (2)	0.008 (2)	0.003 (2)
C13	0.039 (3)	0.054 (3)	0.043 (3)	−0.005 (2)	0.008 (2)	0.000 (2)
C14	0.047 (3)	0.051 (3)	0.053 (3)	−0.002 (2)	0.011 (3)	0.001 (2)
C15	0.075 (4)	0.066 (4)	0.069 (4)	0.004 (3)	0.022 (3)	0.021 (3)
C16	0.110 (6)	0.084 (5)	0.059 (4)	0.009 (4)	0.033 (4)	0.025 (3)
C17	0.088 (5)	0.091 (5)	0.051 (3)	0.003 (4)	0.032 (3)	0.001 (3)
C18	0.058 (4)	0.059 (4)	0.061 (3)	0.004 (3)	0.012 (3)	−0.010 (3)
C19	0.052 (3)	0.047 (3)	0.067 (4)	0.007 (3)	0.005 (3)	−0.002 (3)
C20	0.060 (4)	0.044 (3)	0.052 (3)	0.007 (3)	0.004 (3)	0.001 (2)
C21	0.064 (4)	0.075 (4)	0.100 (5)	0.004 (3)	0.014 (4)	−0.025 (4)
C22	0.105 (6)	0.076 (5)	0.101 (5)	0.026 (4)	0.002 (5)	−0.041 (4)
C23	0.067 (4)	0.069 (4)	0.088 (5)	−0.002 (3)	0.004 (4)	−0.016 (4)
C24	0.054 (4)	0.055 (3)	0.079 (4)	0.005 (3)	0.016 (3)	−0.017 (3)
C25	0.050 (3)	0.048 (3)	0.050 (3)	−0.005 (2)	0.005 (3)	0.001 (2)
C26	0.045 (3)	0.039 (3)	0.050 (3)	−0.001 (2)	0.007 (2)	−0.005 (2)
C27	0.048 (3)	0.060 (4)	0.073 (4)	−0.002 (3)	0.014 (3)	0.012 (3)
C28	0.053 (4)	0.086 (4)	0.084 (4)	−0.003 (3)	0.007 (3)	0.039 (4)
C29	0.048 (4)	0.061 (4)	0.077 (4)	0.005 (3)	0.009 (3)	0.020 (3)
C30	0.050 (4)	0.054 (3)	0.058 (3)	0.003 (3)	0.015 (3)	0.012 (3)
N1	0.053 (3)	0.047 (2)	0.046 (2)	0.001 (2)	0.008 (2)	0.003 (2)
N2	0.051 (3)	0.046 (3)	0.052 (3)	−0.001 (2)	0.014 (2)	0.000 (2)
N3	0.043 (3)	0.052 (3)	0.042 (2)	0.0028 (19)	0.0033 (19)	−0.002 (2)
N4	0.044 (3)	0.050 (3)	0.054 (3)	0.004 (2)	0.011 (2)	0.000 (2)
N5	0.054 (3)	0.057 (3)	0.065 (3)	0.008 (2)	0.011 (2)	0.013 (2)
N6	0.057 (3)	0.049 (3)	0.076 (3)	−0.002 (2)	0.008 (3)	−0.016 (2)
Cl1	0.0595 (9)	0.0634 (9)	0.0718 (9)	−0.0083 (7)	−0.0024 (7)	0.0051 (7)
Cl2	0.0494 (9)	0.0694 (10)	0.1084 (12)	0.0091 (7)	0.0093 (8)	0.0271 (9)

### Geometric parameters (Å, °)

**Table:**

Cu1—N4	1.940 (4)	C14—N2	1.423 (6)
Cu1—N6^i^	2.079 (5)	C15—C16	1.367 (8)
Cu1—Cl1	2.1836 (16)	C15—H15	0.9300
Cu2—N1	1.942 (4)	C16—C17	1.387 (8)
Cu2—N5^ii^	2.116 (4)	C16—H16	0.9300
Cu2—Cl2	2.1672 (16)	C17—C18	1.378 (7)
C1—N4	1.318 (6)	C17—H17	0.9300
C1—N3	1.363 (6)	C18—H18	0.9300
C1—C8	1.502 (7)	C19—N2	1.462 (6)
C2—C7	1.375 (7)	C19—C20	1.510 (7)
C2—N4	1.379 (6)	C19—H19A	0.9700
C2—C3	1.391 (7)	C19—H19B	0.9700
C3—C4	1.380 (7)	C20—C21	1.372 (8)
C3—N3	1.385 (6)	C20—C24	1.429 (7)
C4—C5	1.390 (8)	C21—C22	1.374 (9)
C4—H4	0.9300	C21—H21	0.9300
C5—C6	1.379 (8)	C22—C23	1.408 (9)
C5—H5	0.9300	C22—H22	0.9300
C6—C7	1.376 (8)	C23—N6	1.324 (7)
C6—H6	0.9300	C23—H23	0.9300
C7—H7	0.9300	C24—N6	1.319 (7)
C8—C9	1.535 (7)	C24—H24	0.9300
C8—H8A	0.9700	C25—N3	1.455 (6)
C8—H8B	0.9700	C25—C26	1.511 (7)
C9—C10	1.517 (6)	C25—H25A	0.9700
C9—H9A	0.9700	C25—H25B	0.9700
C9—H9B	0.9700	C26—C30	1.361 (7)
C10—C11	1.526 (7)	C26—C27	1.373 (7)
C10—H10A	0.9700	C27—C28	1.377 (8)
C10—H10B	0.9700	C27—H27	0.9300
C11—C12	1.485 (6)	C28—C29	1.355 (7)
C11—H11A	0.9700	C28—H28	0.9300
C11—H11B	0.9700	C29—N5	1.335 (6)
C12—N1	1.332 (6)	C29—H29	0.9300
C12—N2	1.357 (6)	C30—N5	1.338 (6)
C13—C18	1.379 (6)	C30—H30	0.9300
C13—C14	1.399 (7)	N5—Cu2^ii^	2.116 (4)
C13—N1	1.414 (6)	N6—Cu1^i^	2.079 (5)
C14—C15	1.387 (7)		
			
N4—Cu1—N6^i^	111.23 (18)	C18—C17—C16	121.3 (5)
N4—Cu1—Cl1	141.19 (13)	C18—C17—H17	119.3
N6^i^—Cu1—Cl1	107.12 (13)	C16—C17—H17	119.3
N1—Cu2—N5^ii^	108.71 (17)	C17—C18—C13	118.0 (5)
N1—Cu2—Cl2	142.93 (13)	C17—C18—H18	121.0
N5^ii^—Cu2—Cl2	107.36 (13)	C13—C18—H18	121.0
N4—C1—N3	113.3 (4)	N2—C19—C20	111.4 (4)
N4—C1—C8	123.9 (5)	N2—C19—H19A	109.3
N3—C1—C8	122.8 (5)	C20—C19—H19A	109.3
C7—C2—N4	129.4 (5)	N2—C19—H19B	109.3
C7—C2—C3	121.1 (5)	C20—C19—H19B	109.3
N4—C2—C3	109.5 (4)	H19A—C19—H19B	108.0
C4—C3—N3	131.1 (5)	C21—C20—C24	116.3 (5)
C4—C3—C2	122.6 (5)	C21—C20—C19	119.5 (5)
N3—C3—C2	106.3 (4)	C24—C20—C19	124.1 (5)
C3—C4—C5	115.4 (6)	C20—C21—C22	119.2 (6)
C3—C4—H4	122.3	C20—C21—H21	120.4
C5—C4—H4	122.3	C22—C21—H21	120.4
C6—C5—C4	122.0 (6)	C21—C22—C23	120.7 (6)
C6—C5—H5	119.0	C21—C22—H22	119.7
C4—C5—H5	119.0	C23—C22—H22	119.7
C7—C6—C5	122.0 (6)	N6—C23—C22	120.6 (6)
C7—C6—H6	119.0	N6—C23—H23	119.7
C5—C6—H6	119.0	C22—C23—H23	119.7
C2—C7—C6	116.8 (5)	N6—C24—C20	124.2 (5)
C2—C7—H7	121.6	N6—C24—H24	117.9
C6—C7—H7	121.6	C20—C24—H24	117.9
C1—C8—C9	112.9 (4)	N3—C25—C26	112.2 (4)
C1—C8—H8A	109.0	N3—C25—H25A	109.2
C9—C8—H8A	109.0	C26—C25—H25A	109.2
C1—C8—H8B	109.0	N3—C25—H25B	109.2
C9—C8—H8B	109.0	C26—C25—H25B	109.2
H8A—C8—H8B	107.8	H25A—C25—H25B	107.9
C10—C9—C8	110.5 (4)	C30—C26—C27	117.0 (5)
C10—C9—H9A	109.5	C30—C26—C25	121.1 (5)
C8—C9—H9A	109.5	C27—C26—C25	121.8 (5)
C10—C9—H9B	109.5	C26—C27—C28	120.1 (5)
C8—C9—H9B	109.5	C26—C27—H27	120.0
H9A—C9—H9B	108.1	C28—C27—H27	120.0
C9—C10—C11	112.7 (4)	C29—C28—C27	118.6 (5)
C9—C10—H10A	109.0	C29—C28—H28	120.7
C11—C10—H10A	109.0	C27—C28—H28	120.7
C9—C10—H10B	109.0	N5—C29—C28	123.0 (5)
C11—C10—H10B	109.0	N5—C29—H29	118.5
H10A—C10—H10B	107.8	C28—C29—H29	118.5
C12—C11—C10	113.0 (4)	N5—C30—C26	124.3 (5)
C12—C11—H11A	109.0	N5—C30—H30	117.8
C10—C11—H11A	109.0	C26—C30—H30	117.8
C12—C11—H11B	109.0	C12—N1—C13	106.8 (4)
C10—C11—H11B	109.0	C12—N1—Cu2	124.7 (3)
H11A—C11—H11B	107.8	C13—N1—Cu2	128.5 (3)
N1—C12—N2	111.8 (4)	C12—N2—C14	107.5 (4)
N1—C12—C11	123.4 (4)	C12—N2—C19	127.1 (4)
N2—C12—C11	124.8 (5)	C14—N2—C19	124.1 (4)
C18—C13—C14	120.0 (5)	C1—N3—C3	105.7 (4)
C18—C13—N1	131.5 (5)	C1—N3—C25	128.3 (4)
C14—C13—N1	108.5 (4)	C3—N3—C25	125.5 (4)
C15—C14—C13	122.0 (5)	C1—N4—C2	105.2 (4)
C15—C14—N2	132.5 (5)	C1—N4—Cu1	131.3 (3)
C13—C14—N2	105.5 (4)	C2—N4—Cu1	123.3 (3)
C16—C15—C14	116.9 (5)	C29—N5—C30	117.0 (5)
C16—C15—H15	121.5	C29—N5—Cu2^ii^	120.4 (4)
C14—C15—H15	121.5	C30—N5—Cu2^ii^	121.7 (4)
C15—C16—C17	121.7 (5)	C24—N6—C23	118.9 (5)
C15—C16—H16	119.1	C24—N6—Cu1^i^	119.8 (4)
C17—C16—H16	119.1	C23—N6—Cu1^i^	121.0 (4)
			
C7—C2—C3—C4	1.2 (8)	C18—C13—N1—C12	179.1 (5)
N4—C2—C3—C4	−179.0 (5)	C14—C13—N1—C12	−0.9 (5)
C7—C2—C3—N3	−179.8 (5)	C18—C13—N1—Cu2	−3.1 (8)
N4—C2—C3—N3	0.0 (5)	C14—C13—N1—Cu2	176.9 (3)
N3—C3—C4—C5	178.9 (5)	N5^ii^—Cu2—N1—C12	57.7 (4)
C2—C3—C4—C5	−2.4 (8)	Cl2—Cu2—N1—C12	−108.5 (4)
C3—C4—C5—C6	0.8 (10)	N5^ii^—Cu2—N1—C13	−119.7 (4)
C4—C5—C6—C7	2.2 (11)	Cl2—Cu2—N1—C13	74.1 (5)
N4—C2—C7—C6	−178.0 (6)	N1—C12—N2—C14	−1.1 (6)
C3—C2—C7—C6	1.8 (8)	C11—C12—N2—C14	177.4 (5)
C5—C6—C7—C2	−3.4 (10)	N1—C12—N2—C19	−168.9 (5)
N4—C1—C8—C9	84.5 (6)	C11—C12—N2—C19	9.6 (8)
N3—C1—C8—C9	−94.6 (6)	C15—C14—N2—C12	178.7 (6)
C1—C8—C9—C10	177.0 (5)	C13—C14—N2—C12	0.5 (5)
C8—C9—C10—C11	179.2 (4)	C15—C14—N2—C19	−13.0 (9)
C9—C10—C11—C12	−164.8 (5)	C13—C14—N2—C19	168.7 (5)
C10—C11—C12—N1	61.0 (7)	C20—C19—N2—C12	79.5 (6)
C10—C11—C12—N2	−117.3 (6)	C20—C19—N2—C14	−86.5 (6)
C18—C13—C14—C15	1.8 (8)	N4—C1—N3—C3	−0.8 (5)
N1—C13—C14—C15	−178.2 (5)	C8—C1—N3—C3	178.3 (4)
C18—C13—C14—N2	−179.7 (4)	N4—C1—N3—C25	−173.0 (4)
N1—C13—C14—N2	0.2 (5)	C8—C1—N3—C25	6.2 (8)
C13—C14—C15—C16	−2.3 (9)	C4—C3—N3—C1	179.4 (6)
N2—C14—C15—C16	179.7 (6)	C2—C3—N3—C1	0.5 (5)
C14—C15—C16—C17	1.5 (10)	C4—C3—N3—C25	−8.2 (8)
C15—C16—C17—C18	−0.1 (11)	C2—C3—N3—C25	172.9 (4)
C16—C17—C18—C13	−0.5 (9)	C26—C25—N3—C1	77.3 (6)
C14—C13—C18—C17	−0.4 (8)	C26—C25—N3—C3	−93.5 (5)
N1—C13—C18—C17	179.7 (5)	N3—C1—N4—C2	0.8 (5)
N2—C19—C20—C21	−158.4 (5)	C8—C1—N4—C2	−178.4 (5)
N2—C19—C20—C24	24.6 (7)	N3—C1—N4—Cu1	−176.0 (3)
C24—C20—C21—C22	0.1 (9)	C8—C1—N4—Cu1	4.9 (7)
C19—C20—C21—C22	−177.1 (6)	C7—C2—N4—C1	179.3 (5)
C20—C21—C22—C23	−1.5 (11)	C3—C2—N4—C1	−0.4 (5)
C21—C22—C23—N6	0.3 (11)	C7—C2—N4—Cu1	−3.6 (7)
C21—C20—C24—N6	2.8 (9)	C3—C2—N4—Cu1	176.7 (3)
C19—C20—C24—N6	179.9 (5)	N6^i^—Cu1—N4—C1	63.3 (5)
N3—C25—C26—C30	48.3 (6)	Cl1—Cu1—N4—C1	−126.0 (4)
N3—C25—C26—C27	−127.6 (5)	N6^i^—Cu1—N4—C2	−113.0 (4)
C30—C26—C27—C28	0.4 (8)	Cl1—Cu1—N4—C2	57.7 (5)
C25—C26—C27—C28	176.4 (5)	C28—C29—N5—C30	−0.6 (9)
C26—C27—C28—C29	−1.6 (9)	C28—C29—N5—Cu2^ii^	−170.4 (5)
C27—C28—C29—N5	1.7 (10)	C26—C30—N5—C29	−0.7 (8)
C27—C26—C30—N5	0.8 (8)	C26—C30—N5—Cu2^ii^	169.0 (4)
C25—C26—C30—N5	−175.2 (5)	C20—C24—N6—C23	−4.1 (9)
N2—C12—N1—C13	1.2 (6)	C20—C24—N6—Cu1^i^	−177.5 (4)
C11—C12—N1—C13	−177.3 (5)	C22—C23—N6—C24	2.5 (9)
N2—C12—N1—Cu2	−176.7 (3)	C22—C23—N6—Cu1^i^	175.8 (5)
C11—C12—N1—Cu2	4.8 (7)		

Symmetry codes: (i) −*x*+2, −*y*+2, −*z*; (ii) −*x*+1, −*y*+2, −*z*.
